# Ku–DNA binding inhibitors modulate the DNA damage response in response to DNA double-strand breaks

**DOI:** 10.1093/narcan/zcad003

**Published:** 2023-02-06

**Authors:** Pamela L Mendoza-Munoz, Navnath S Gavande, Pamela S VanderVere-Carozza, Katherine S Pawelczak, Joseph R Dynlacht, Joy E Garrett, John J Turchi

**Affiliations:** Department of Medicine, Indiana University School of Medicine, Indianapolis, IN 46202, USA; Department of Pharmaceutical Sciences, Wayne State University College of Pharmacy and Health Sciences, Detroit, MI 48201-2417, USA; Molecular Therapeutics Program, Barbara Ann Karmanos Cancer Institute, Wayne State University, Detroit, MI 48201-2417, USA; Department of Medicine, Indiana University School of Medicine, Indianapolis, IN 46202, USA; NERx Biosciences. Indianapolis, IN 46202, USA; Department of Radiation Oncology, Indiana University School of Medicine, Indianapolis, IN 46202, USA; Department of Radiation Oncology, Indiana University School of Medicine, Indianapolis, IN 46202, USA; Department of Medicine, Indiana University School of Medicine, Indianapolis, IN 46202, USA; NERx Biosciences. Indianapolis, IN 46202, USA; Department of Biochemistry and Molecular Biology, Indiana University, School of Medicine, Indianapolis, IN 46202, USA

## Abstract

The DNA-dependent protein kinase (DNA-PK) plays a critical role in the DNA damage response (DDR) and non-homologous end joining (NHEJ) double-strand break (DSB) repair pathways. Consequently, DNA-PK is a validated therapeutic target for cancer treatment in certain DNA repair-deficient cancers and in combination with ionizing radiation (IR). We have previously reported the discovery and development of a novel class of DNA-PK inhibitors with a unique mechanism of action, blocking the Ku 70/80 heterodimer interaction with DNA. These Ku–DNA binding inhibitors (Ku-DBi's) display nanomolar activity *in vitro*, inhibit cellular DNA-PK, NHEJ-catalyzed DSB repair and sensitize non-small cell lung cancer (NSCLC) cells to DSB-inducing agents. In this study, we demonstrate that chemical inhibition of the Ku–DNA interaction potentiates the cellular effects of bleomycin and IR via p53 phosphorylation through the activation of the ATM pathway. This response is concomitant with a reduction of DNA-PK catalytic subunit (DNA-PKcs) autophosphorylation at S2056 and a time-dependent increase in H2AX phosphorylation at S139. These results are consistent with Ku-DBi's abrogating DNA-PKcs autophosphorylation to impact DSB repair and DDR signaling through a novel mechanism of action, and thus represent a promising anticancer therapeutic strategy in combination with DNA DSB-inducing agents.

## Graphical Abstract

**Graphical Abstract:**
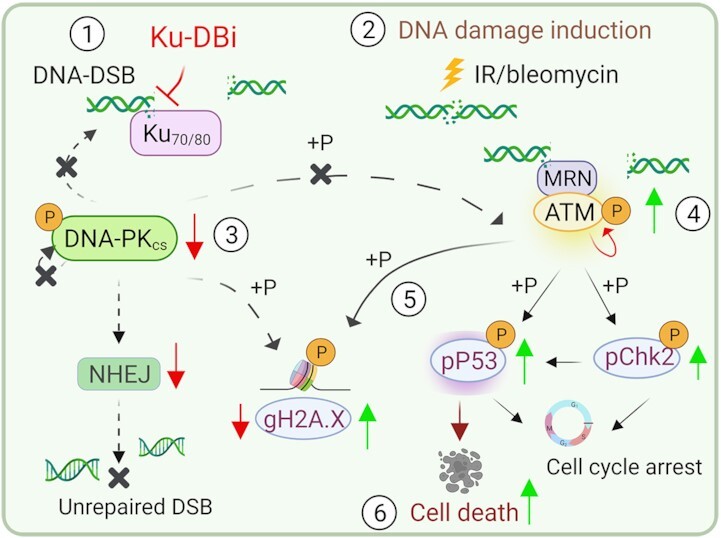
Combined treatment of Ku–DNA binding inhibitors with DNA-DSB inducing agents modulate the activation of DNA damage response via ATM signaling leading to p53 dependent cell death.

## INTRODUCTION

The genome is continually exposed to exogenous and endogenous DNA-damaging agents that alter structure and impact function (1). These events compromise DNA integrity and, if not correctly repaired, lead to genomic instability and cancer development (2). To prevent the accumulation of DNA lesions, cells manage these events through specific DNA damage sensor proteins. These proteins recognize specific structures or changes in the DNA and trigger a complex and coordinated set of events in a process termed the DNA damage response (DDR). DDR initiates cellular signal transduction pathways to ensure cells can respond to all types of DNA damage to promote cell survival through activation of DNA repair pathways and regulation of the cell cycle or triggering of cell death (3–5). The DDR is initiated by the PI-3 kinase-related-kinases (PIKK) ataxia-telangiectasia mutated (ATM), ataxia-telangiectasia and RAD3-related (ATR) and the DNA-dependent protein kinase catalytic subunit (DNA-PKcs), each of which responds to different types of DNA damage or genomic perturbations (6,7). In this context, DNA-PK plays a critical role in sensing DNA double-strand breaks (DSB), the most deleterious lesions in eukaryotic cells (8). DNA-PK is a serine/threonine protein kinase consisting of a 469 kDa catalytic subunit, DNA-PKcs, and the Ku70/80 DNA binding complex. DNA-PK kinase activation requires binding to DNA DSB via direct Ku 70/80 heterodimer-dependent DNA interaction which is essential for DSB repair through the non-homologous end joining (NHEJ) pathway (7,9). DSB-dependent DNA-PK activation is initially mediated by autophosphorylation events at S2056 on the DNA-PKcs subunit (10). In addition, DNA-PKcs can phosphorylate ATM and other downstream targets, as well as be phosphorylated by ATM and ATR at T2609 in certain circumstances (7); (11). For its part, ATM is one of the central kinases involved in the global orchestration of cellular responses to DNA-DSB, being recruited to DSB sites via the MRE11–RAD50–NBS1 (MRN) complex upon activation (12). ATM activation involves rapid intermolecular autophosphorylation events at S1981 (13) and phosphorylation of downstream targets such as p53 and Chk2. Furthermore, ATM activation contributes to the phosphorylation of the histone variant H2AX at S139 (γ-H2AX) which in turn initiates a cascade assembling DDR components at the DSB sites to promote effective DNA damage repair (14).

Many conventional cancer treatments, including ionizing radiation (IR) and some chemotherapeutic drugs, exert their clinical effect via induction of DNA damage to drive cancer cells into apoptosis. Recent advances in our understanding of the mechanisms involved in DDR signaling have opened an exciting array of opportunities to treat human cancers (15–17). Increased expression and deregulation of DNA-PKcs have been associated with the progression of a number of cancers, including multiple myeloma (18) and hepatocellular carcinoma (19). Similarly increased expression of the Ku subunits is associated with radioresistance of thyroid, nasopharynx, oral cavity and cervix tumors (20). Accordingly, DNA-PK is a validated therapeutic target for cancer treatment in certain DNA repair-deficient cancers and in combination with IR or DSB-inducing chemotherapeutic agents (20–24). We have previously reported the discovery and development of a new class of DNA-PK inhibitors with a novel mechanism of action that specifically targets the Ku 70/80 heterodimer interaction with DNA (25). These Ku–DNA binding inhibitors (Ku-DBi's) display nanomolar activity *in vitro*, inhibit cellular DNA-PK and NHEJ-catalyzed DSB repair, and sensitize non-small cell lung cancer (NSCLC) cells to DSB-inducing agents. Here we demonstrate that chemical inhibition of the Ku–DNA interaction mediated by Ku-DBi's potentiates the cellular effects of bleomycin and IR via p53 phosphorylation through the activation of the ATM pathway. In addition, we demonstrate that Ku–DNA binding inhibition differentially impacts cells carrying ATM and/or p53 alterations, which supports the use of Ku-DBi to sensitize cells with different genetic backgrounds through the induction of p53 phosphorylation in combined treatment with DSB-inducing agents.

These data are consistent with Ku-DBi's possessing a novel mechanism that abrogates DNA-PKcs autophosphorylation to impact DSB repair and modulates DDR signaling, and their use represents a promising anticancer therapeutic strategy when used in combination with DNA DSB-inducing agents.

## MATERIALS AND METHODS

### Compounds

All Ku–DNA binding inhibitors were developed and previously reported by our lab (25). Ku-DBi's were dissolved in dimethylsulfoxide (DMSO; Cat: BP231-100, Fisher bioreagents) to a concentration of 10 mM. The DNA-PK catalytic site inhibitor **NU-7441** (Cat: 3712, TOCRIS) was dissolved in DMSO to a concentration of 5 mM. All compounds were stored at room temperature.

### Cell culture

Human non-small cell lung cancer cell lines NCI-H460 (HTB-177™), A549 (CCL-185™) and NCI-H23 (CRL-5800™) were obtained from ATCC®. The H1299 (CRL-5803™) cell line was provided by Dr Catherine Sears (Indiana University, School of Medicine, Indianapolis, IN). The triple negative breast cancer (TNBC) cell line MDA-MB-436 (HTB-130) was obtained from ATCC®. H460, H23 and H1299 cells were cultured in RPMI-1640 medium (RPMI-1640 with l-glutamine. Cat:12-702F, BioWhittaker® Lonza) supplemented with 10% fetal bovine serum and 1× Penicillin Streptomycin (Penicillin Streptomycin solution, 100×. Penicillin (10 000 IU/ml) and Streptomycin (10000 μg/ml). Cat: 30–002-CI, Corning). A549 cells were cultured in DMEM (Dulbecco's modified Eagle's medium, with 4.5 g/l glucose, l-glutamine & sodium pyruvate. Cat: 10-013-CV, Corning) supplemented with 10% fetal bovine serum and 1× Penicillin Streptomycin). MDA-MB-436 cells were cultured in DMEM/F-12 (Dulbecco's modified Eagle's medium, with 4.5 g/l glucose, l-glutamine & sodium pyruvate. Cat: 10-013-CV, Corning, and Ham's F-12, 1× modified with l-glutamine. Cat: 10-080-CV, Corning) supplemented with 10% fetal bovine serum and 1× Penicillin Streptomycin. Cells were plated in wells and incubated at 37°C in 5% CO_2_ for 24 h prior to any treatment. Cells then were treated with 1% DMSO as a vehicle, or Ku-DBi prepared in OptiMEM (Opti-MEM® I 1× with HEPES, 2.4 g/l sodium bicarbonate and l-glutamine. Cat: 31985-070, Gibco), and doses of bleomycin (bleomycin sulfate, cell grade. Cat: J67560-S, Alfa Aesar) or IR (2.5 or 10 Gy) as described in the figure legends. For ATM inhibition assays, NSCLC cells were treated with ATM kinase inhibitor **AZD0156** (Cat: S8375, Selleck Chemicals) as described in the figure legends.

### Cell viability assays

Cell metabolism/viability was assessed by a mitochondrial metabolism assay (CCK-8) kit (Cell counting Kit-8, Cat: CK04, Dojindo Laboratories), as we have previously described (26). This assay is based on the generation of a water-soluble formazan product by cellular dehydrogenases and is proportional to the number of metabolically active cells. Briefly, 3 × 10^3^ cells were seeded in 96-well plates and treated 24 h later as described in the legends. Following 72 h treatment, CCK-8 reagent was added to each well, and cells were incubated for 1–2 h at 37°C. The absorbance of the formed formazan product in each well was measured at 450 nm and compared to vehicle-treated controls to determine percent cell viability. The results presented are the average and SEM of triplicate determinations.

### Clonogenic survival assays

H460 cells were seeded and cultured for 24 h in 24-well plates prior to drug treatment. Cells were treated with either 20 μM Ku-DBi for 2 h, 10 μM bleomycin for 1 h or vehicle, as indicated. All drug and vehicle treatments were performed at a final concentration of 1% DMSO. Then cells were detached with trypsin, plated in 100mm dishes and incubated 37°C in 5% CO_2_ for 11 days. Medium was removed and cells were carefully washed once in phosphate buffered saline (PBS) before fixing and staining in 0.5% crystal violet (Cat: C581-100, Fisher Scientific) supplemented with 6% glutaraldehyde (Glutaraldehyde solution, 25%. Cat: O2957-1, Fisher Scientific) in PBS solution for 1 h at RT. Staining solution was removed and cells washed in water and air-dried. The colonies formed were manually counted and surviving fractions were determined and normalized to control condition treated with vehicle only.

### Incucyte live-cell analysis

2 × 10^3^ NSCLC H460 cells were plated in a 96-well plate in RPMI supplemented with 10% FBS and incubated at 37°C in 5% CO_2_ for 24 h prior to treatments. The medium was replaced with fresh reduced serum medium (OptiMEM) or 10% FBS supplemented RPMI containing vehicle, Ku-DBi (20 μM), bleomycin (1 μM) or the combination. Cell growth was assessed by a 7-day live cell imaging assay (Incucyte S3 Automated Live-Cell Analysis, Sartorius). Images were acquired and phase object confluence percentage was determined every 4 h. Data were analyzed and are presented as the mean and SEM of triplicate determinations.

### Cell irradiation

NSCLC cells were seeded into 24-well plates 18–24 h prior to experiments and the medium was replaced with fresh medium containing either Ku-DBi or vehicle 2 h prior to irradiation. Plates were placed on ice for 10 min prior to irradiation. Exponentially growing cells were then irradiated on ice with 2, 5 or 10 Gy of 160 kVp x-rays using a Precision X-ray machine (North Branford, CT) at a dose rate of 0.687 Gy/min. Radiation dosimetry measurements were performed using a Farmer-type ionization chamber (PTW Model N30013, Freiburg, Germany) in conjunction with a Keithley electrometer (Model K602, Cleveland, Ohio). After irradiation, cells were incubated for 1 h at 37°C before processing.

### Immunofluorescence microscopy

Cells were seeded on cover glass in a 24-well plate and cultured overnight prior to treatment. Cells were pre-incubated with the compound or vehicle for the indicated time prior to treatment with 10 μM bleomycin for 1 h at 37°C. After bleomycin treatment, cells were washed in PBS, fixed with 4% cold paraformaldehyde for 20 min, permeabilized in 0.1% Triton X-100 for 10 min, washed and incubated in blocking solution (3% BSA, 5% Goat serum in PBS) for 30 min at room temperature. Primary antibodies against phospho-DNA-PKcs S2056 (Cat: ab124918, Abcam), phospho-H2AX S139 (Cat: 613401, BioLegend), or phospho-ATM S1981 (Cat: ab81292, Abcam) prepared at 1:100 or 1:200 dilution in blocking solution were added to cells and incubated overnight at 4°C. Then an appropriate Alexa Fluor-488 or -594 conjugated secondary antibody [Goat anti-Rabbit IgG (H+L) Cross-Adsorbed Secondary Antibody, Alexa Fluor-488 (Cat: A-11008, Invitrogen), or Goat anti-Mouse IgG (H+L) Cross-Adsorbed Secondary Antibody, Alexa Fluor-594 (Cat: A-11005, Invitrogen) prepared at 1:200 dilution in blocking solution was added to cells and then incubated for 2 h at room temperature. The cover glass with cells were mounted on slides in antifade mounting medium with the DNA stain DAPI (VectaShield HardSet. Cat: H-1500, Vector Laboratories). Images were captured digitally using the inverted multi-channel fluorescence EVOS FL Auto 2 Imaging System (Invitrogen) using 10× or 60×-immersion magnification. The fluorescence intensity for red and green channel was quantified using the ImageJ FIJI extended version. First, a binary mask was created from each 10× DAPI inverted color image for DAPI-positive regions in 200 nuclei per field and were defined as regions of interest (ROI). The criteria for ROI selection included the Huang auto thresholding method, and cell exclusion on edges. Particles were analyzed in a size range of 200–500-pixel units and 0.00–1.00 circularity. Once the binary mask was created, it was opened and applied on the green or red channel, and raw integrated density was measured. DAPI, phospho-DNA-PKcs S2056, phospho-ATM S1980 and phospho-H2AX S139 signals were determined in blue, green, or red channels, respectively.

### Protein extraction and western blotting

Cells were cultured in 6-well plates overnight prior to pre-treatment with vehicle, 20 μM Ku-DBi's or 10 μM **NU-7441** for 1 or 2 h at 37°C. Cells were then treated with 10 μM bleomycin or irradiated with 5 Gy of X-rays. After treatment, cells were incubated on ice and washed in cold PBS and then lysed with RIPA buffer (50 mM Tris, pH 8.0, 150 mM sodium chloride, 1% NP-40, 0.5% sodium deoxycholate, 0.1% SDS, 1 mM EDTA) containing 1× protease and phosphatase inhibitors [Halt™ Protease and phosphatase inhibitor, single-use cocktail (100×). Cat: 78443, Thermo Scientific] added before use. The lysates were sonicated, centrifuged for 20 min at 14 000 rpm, and the supernatants were collected, and the protein content was quantified using a BCA protein assay kit (Pierce™ BCA protein assay kit. Cat: 23227, Thermo Scientific). 20–30 μg of protein lysates were separated by SDS-PAGE (4–20% Mini Protean TGX Gels, BioRad), and wet-transferred onto PVDF membrane (Cat: 1620177, Bio-Rad). Membranes were blocked for 1 h in 1× TBS with 0.5% Tween and 2% non-fat dried milk and probed with the following primary antibodies against phospho-DNA PKcs (S2056) (Cat: ab124918, Abcam), phospho-DNA PKcs (T2609) (Cat: ab4194, Abcam), total DNA-PKcs (Cat: sc-5282, Santa Cruz Biotechnology), γ-H2AX pS139 (Cat: 613401, BioLegend), phospho-ATM (Ser1981) (Cat: ab81292, Abcam), total ATM (Cat: 2873, Cell Signaling), phospho-p53 (S15) (Cat: 9284 S, Cell signaling), phospho-Chk1 (Ser345) (Cat: 2348, Cell signaling), phospho-Chk2 (Thr68) (Cat: 2661, Cell Signaling) and GAPDH (Cat: MA-15738, Thermo Fisher), diluted in TBST with 2% BSA and 0.02% NaN_3_. Goat Anti-Rabbit or Anti-Mouse IgG (H+L)-HRP conjugate (Cat: 170-6515 or 170-6516, Bio-Rad) were used as secondary antibodies to detect these proteins.

### Statistical analysis

Ordinary one-way ANOVA analysis multiple comparisons were performed using GraphPad Prism software (v.9.3.1).

## RESULTS

### Inhibition of Ku-DNA binding potentiates the cellular effects of bleomycin without impacting cellular survival

**Figure 1.**
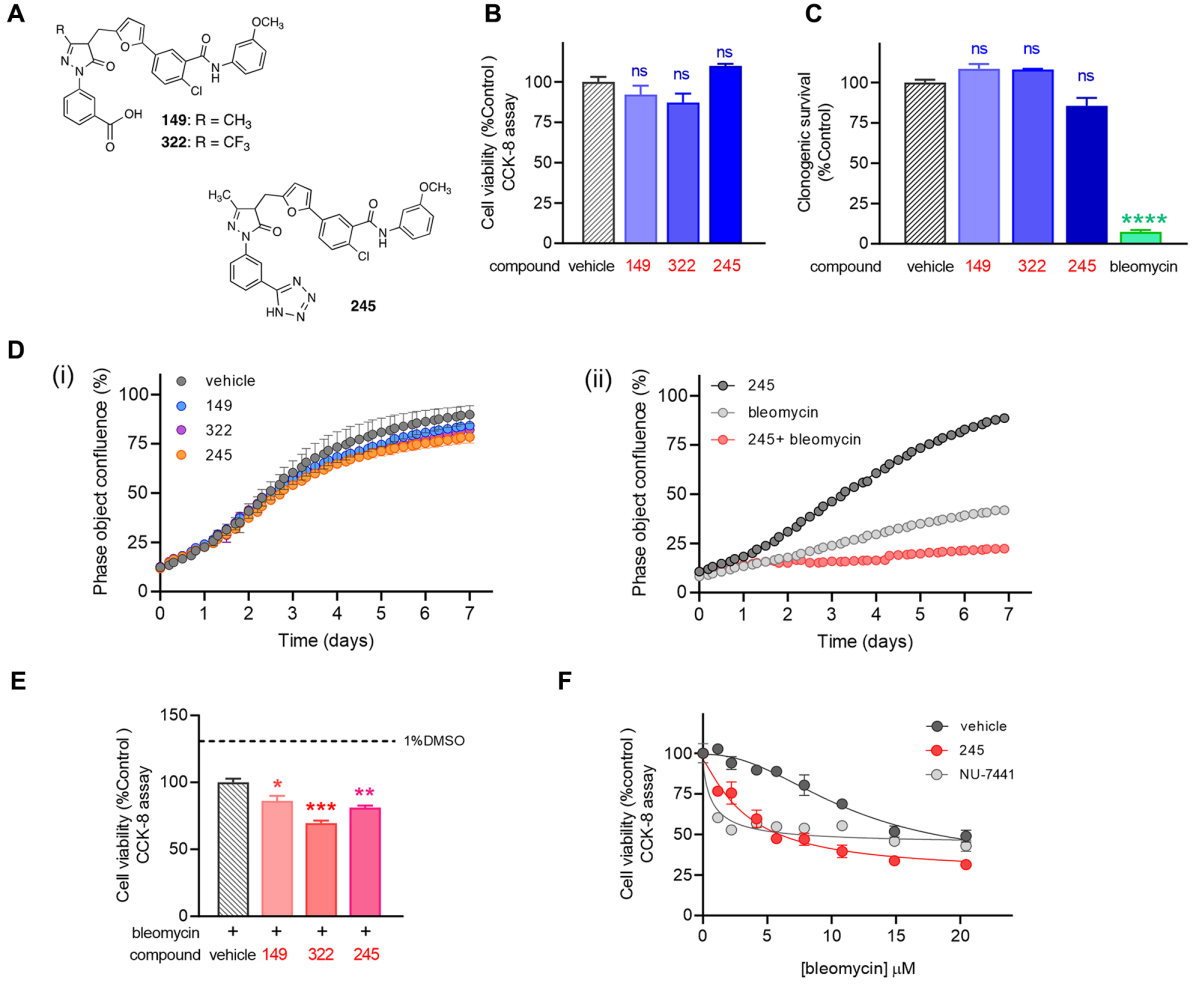
Ku–DNA inhibition does not affect the cellular viability, but it does potentiate the cellular effects of bleomycin. (**A**) Chemical structures of Ku-DBi's. (**B**) NSCLC H460 cells were plated and treated 20 μM Ku-DBi as single agents or 1% DMSO as vehicle and incubated for 72 h after which cell viability was determined by CCK-8 assay. (**C**) Clonogenic survival assay for single agent treatment. H460 cells were incubated with 20 μM Ku-DBi, 1 h 10 μM bleomycin or vehicle. Once treatment was completed, the medium was replaced by supplemented media and cells were detached with trypsin, re-plated, and incubated for 11 days. Colonies formed were fixed, stained, and counted. Data are presented as the mean and SEM of triplicate determinations. (**D**) Incucyte Live cell analysis. NSCLC H460 cells were incubated for 7 days with 20 μM Ku-DBi **149**, **322** and **245** or vehicle (i), or 1 μM bleomycin, 20 μM **245** or combined treatment (ii). Cell growth was assessed over 7 days in a live cell analysis. Images were acquired, and confluence percentage was determined every 4 h. Data are presented as the mean and SEM of triplicate determinations. (**E**) Effect of Ku–DNA binding inhibition on the cellular sensitization to bleomycin. H460 cells were pre-incubated for 24 h with 20 μM Ku-DBi or vehicle followed by 10 μM bleomycin treatment. Cells were incubated for 72 h after which cell viability was determined by CCK-8 assay. Data are presented as the mean and SEM of triplicate determinations. ****P < 0.0001, ***P = 0.0001, **P = 0.0025, *P = 0.0150 as calculated by one-way ANOVA multiple comparisons test. (**F**) Cellular sensitization to bleomycin by Ku-DBi **245**. H460 cells were plated and treated with vehicle, 20 μM **245** or 10 μM **NU-7441** and increasing concentrations of bleomycin for 48 h after which cell viability was determined by CCK-8. Data are presented as the mean and SEM of triplicate determinations.

We previously reported the discovery and development of a new class of DNA-PK inhibitors (Figure 1A) whose mechanism of action involves blocking the Ku–DNA interaction (25). To understand how inhibition of Ku–DNA binding influences cellular survival, we first assessed the effect of Ku-DBi single agent treatment on cell viability in H460 cells. Cells were incubated with Ku-DBi (20 μM) or 1% DMSO vehicle prepared in OptiMEM, and CCK-8 assays were performed 72 h after treatments (Figure 1B). Cell viability was determined as a percentage of control. The results showed that 72 h following a Ku-DBi treatment, cell viability was not significantly affected when compared to the DMSO control (Figure 1B). This is expected as short-term genetic depletion of Ku protein levels has been reported to have minimal effect on cellular viability (27); (28). These results were confirmed in a clonogenic survival assay where short-term Ku inhibitor treatment did not display a significant effect on cell viability (Figure 1C).

Consistent with previous work demonstrating the requirement of Ku in long term culture of human cells (29), we would expect Ku loss or inhibition induced by Ku-DBi's to affect the cellular viability over time. To explore this, we analyzed the effect of Ku inhibition using our Ku-DBi's as single agents on the H460 cells growth over a 7-day period with the Incucyte live-cell imaging analysis (Figure 1D). To perform this assay, cells were treated with Ku-DBi or vehicle, and cell growth was assessed over 7 days in a live cell analysis. We observed that Ku-DBi treatments did not induce notable changes in cell growth up to 72 h, when compared to the vehicle control condition. However, continued incubation did result in reduced confluency over the following 4 days. Ku-DBi **245** displayed the largest effect among all the Ku-DBi's tested, reaching about 78% cellular confluence at the end of the assay compared to 90% confluence for the vehicle control (Figure 1D, panel i). This could be a phenomenon occurring due to a lengthening of the cell cycle or stalled growth/senescence in the short term and cell death in the long term. Statistical analysis from Incucyte experiments showed a significant reduction in cellular confluence in Ku-DBi **245** treatment at the end of the assay; however, no statistical differences in the overall rate growth were detected (Table S1). Incucyte live imaging throughout the assay allowed us to look at these cells, and determine similar cellular size was observed for both Ku-DBi treated and control conditions over the time. Nevertheless, a moderate difference between Ku-DBi-treated cells proliferation and the untreated condition from day 3 (supplemental Movies S1 and S2). This modest increase in doubling time could explain the lower cellular confluence obtained after 7 day incubation. These observations suggest that Ku-DBi treatment results in decreased proliferation but does not lead to cell death. These results are consistent with data demonstrating that inhibition of DNA-PK catalytic subunit with **NU-7441** reduced the rate of cell proliferation in prostate cancer cells (30). These data together, suggest that decreased proliferation occurs in cells independent of the mechanism of DNA-PK inhibition.

To assess the impact of Ku-DBi treatment on the cellular effects of bleomycin we performed combination studies using Incucyte live-cell imaging assays (Figure 1D, panel ii) and CCK-8 assays (Figure 1E, F). Cells treated with Ku-DBi **245** in combination with bleomycin revealed a significant potentiation of sensitivity to bleomycin (Figure 1D, panel ii; Supplemental Movie S3 to S5). These results were confirmed with each of the Ku-DBis tested (Figure 1E). The results obtained showed a modest but statistically significant reduction for combined treatment with bleomycin when H460 cells were pre-incubated with Ku-DBi's **245** and **322** eliciting the greatest effect. Titration of bleomycin at a fixed **245** concentration demonstrated a dose-dependent effect (Figure 1F) and together, these results demonstrate that Ku-DBi's do not affect the short-term cellular viability as single agent but can potentiate the cellular effect of DNA-damaging agents to induce cellular death.

### Ku–DNA binding inhibitors impact cellular response to the radiomimetic DNA-DSB inducing agent bleomycin through inhibition of DNA-PKcs autophosphorylation events at S2056

**Figure 2.**
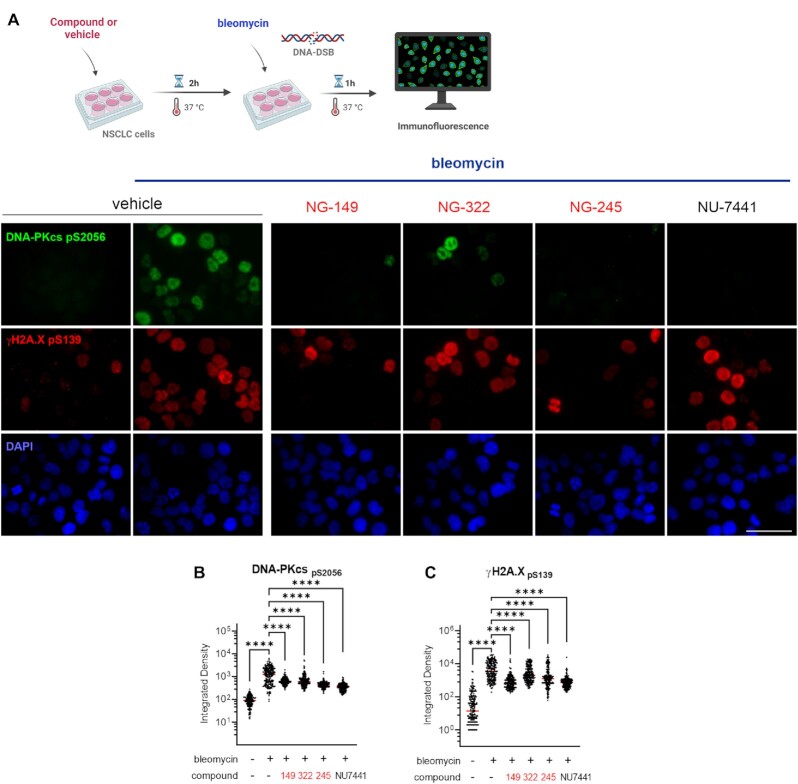
Impact of novel Ku–DNA binding inhibitors on S2056 autophosphorylation in response to bleomycin. (**A**) Schematic representation of bleomycin treatment and compounds or vehicle pre-incubation used in this assay, created in *BioRender.com* (Top). H460 cells were incubated for 2 h with vehicle, Ku-DBi or **NU-7441** and 1 h bleomycin treatment was applied prior to fixing the cells for immunofluorescence detection. Immunofluorescence staining of DNA-PKcs p(S2056) (green), γ-H2AX (pS139) (red), and DAPI nuclear staining (blue) following treatment (Bottom). Sb: 10 μm. (**B**) Quantification of integrated fluorescence intensity of DNA-PKcs and (B) γ-H2AX foci (**C**). Data are presented as the mean and SEM of triplicate determinations. ****P < 0.0001 as calculated by one-way ANOVA with Tukey's multiple comparisons test.

Having established that inhibition of Ku–DNA binding activity increases cell death induced by bleomycin, we sought to determine the mechanisms involved in potentiating the cellular response to DNA damage. Considering the essential role of Ku–DNA binding in DNA-PK activation, we assessed autophosphorylation of the catalytic subunit of DNA-PK (DNA-PKcs) at S2056 in response to treatment with the radiomimetic agent bleomycin and how this is impacted by Ku-DBi treatment. We assessed DNA-PKcs autophosphorylation and DDR signaling by 2 h pre-incubation with either 1% DMSO vehicle, the DNA-PK catalytic subunit inhibitor NU7441 or Ku-DBi, followed by the addition of 10 μM bleomycin for 1 h (Figure 2A, top). Immediately after treatments, cells were fixed, and phosphorylation of DNA-PKcs at S2056 and γ-H2AX levels as a marker of DNA damage (31) were assessed by immunofluorescence (IF) detection. Images acquired by fluorescence microscopy were quantified to analyze their integrated fluorescence intensity. The results showed each Ku-DBi resulted in a decrease in the DNA-PKcs autophosphorylation at S2056 and a reduction in γ-H2AX as assessed by phosphorylation at S139, demonstrating an early inhibition of the DDR (Figure 2A, bottom). Quantification of integrated fluorescence intensity confirmed these results, demonstrating a significant reduction in autophosphorylation of DNA-PKcs and H2AX phosphorylation for each Ku-DBi tested (Figures 2B and C). These results demonstrate that blocking the Ku–DNA interaction reduces DNA-PK autophosphorylation and DDR signaling in response to bleomycin-induced DNA damage. Statistically significant reductions in DNA-PKcs autophosphorylation were observed for each of the Ku-DBi's with only modest differences between the individual inhibitors.

### Ku-DBi's block DNA-DSB dependent DNA-PKcs autophosphorylation, resulting in an enhancement of cellular radiopotentiation

**Figure 3.**
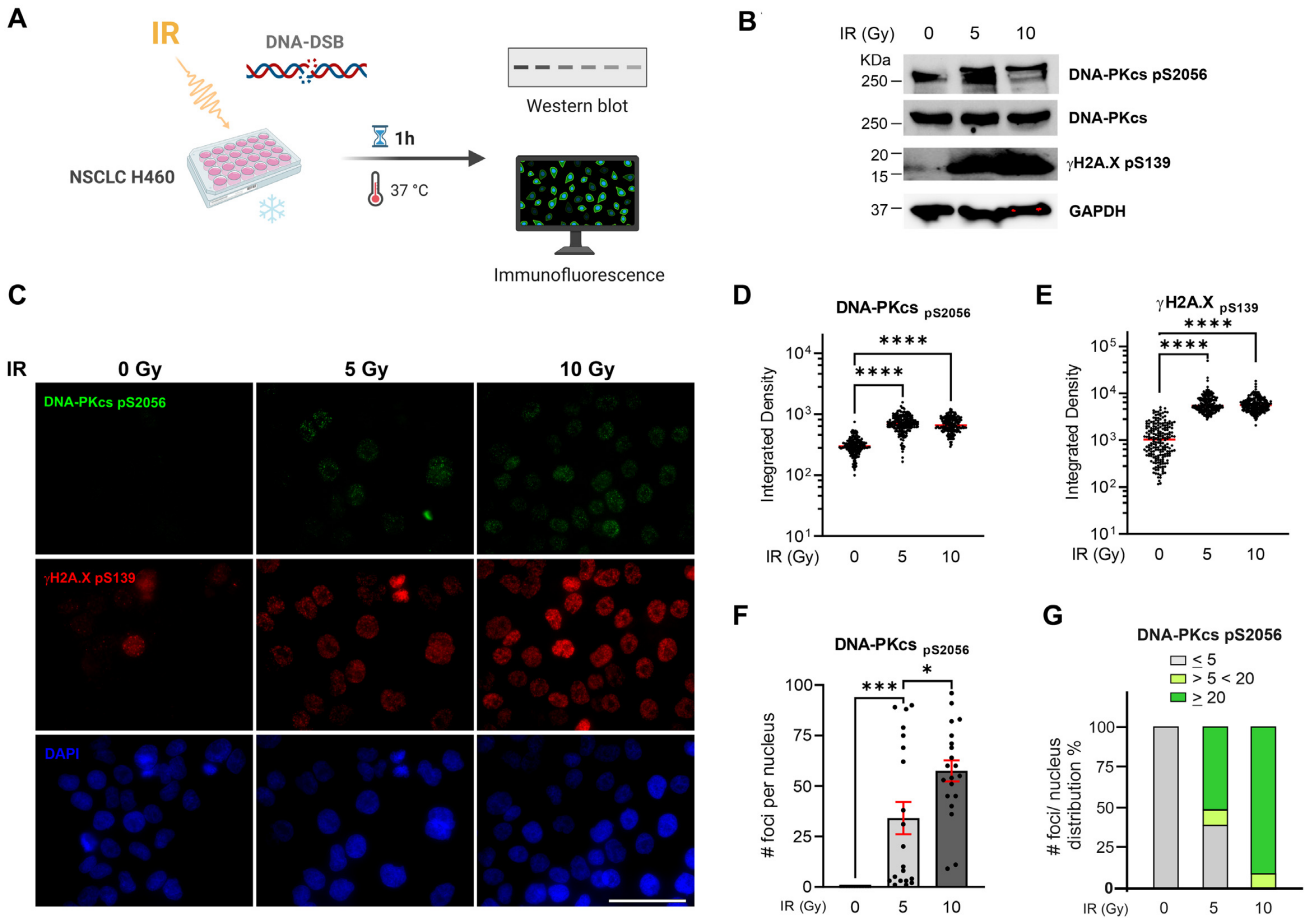
Impact of ionizing radiation on the DNA-PKcs autophosphorylation at S2056. (**A**) Increase in S2056 autophosphorylation in response to IR. Schematic representation of treatment, created in *BioRender.com*. (**B**) Western blot analysis from H460 cells extracts (**C**) IF staining of DNA-PKcs p(S2056) (green), γ-H2AX (pS139) (red), and DAPI nuclear staining (blue). Sb: 10 μm. (**D**) Quantitation of integrated fluorescence intensity of DNA-PKcs foci. (**E**) Quantitation of integrated fluorescence intensity of γ-H2AX foci. Data are presented as the mean and SEM of triplicate determinations. ****P <0.0001 as calculated by one-way ANOVA with Tukey's multiple comparisons test. (**F**) Quantification of DNA-PKcs foci number per nucleus. (**G**) Percentage of distribution of DNA-PKcs foci per nucleus. Data are presented as the mean and SEM of triplicate determinations. ***P = 0.0001, *P = 0.0103 as calculated by one-way ANOVA with Tukey's multiple comparisons test.

Having determined that Ku-DBi dependent DDR inhibition does not influence short-term cell viability and that Ku-DBi's mechanism of action is concomitant with the abrogation of DNA-PKcs autophosphorylation at S2056 after DNA-DSB with bleomycin, along with our previously reported data on Ku-DBi effect on cellular sensitization (25), we wanted to evaluate S2056 autophosphorylation in response to ionizing radiation (IR). H460 cells were exposed to 0, 5 or 10 Gy of X-rays and allowed to recover for 1 h at 37°C before preparing protein lysates for western blot detection or fixing cells for IF detection of DNA-PKcs phosphorylation and γ-H2AX (Figure 3A). Western blot results showed a noticeable increase in the levels of phospho-DNA-PKcs S2056 and γ-H2AX after exposure to either 5 Gy or 10 Gy followed by 1 h recovery, confirming the activation of DNA-PK and DDR pathways in response to DNA-DSB induction (Figure 3B). Results obtained from IF assays showed a marked increase in the phosphorylation of DNA-PKcs and γ-H2AX which was consistent with the results from the western blot detection (Figure 3C). The quantification of integrated fluorescence intensity showed no significant differences between 5 Gy and 10 Gy doses with regard to DSB induction and DNA damage increased levels (Figure 3D and E). These results showed that 5 Gy is a sufficient dose to induce DNA damage that allow us to detect changes in DNA-PKcs phosphorylation events at S2056 and γ-H2AX through IF or Western blot. Therefore, we chose the IR dose of 5 Gy to proceed with our next series of experiments.

**Figure 4.**
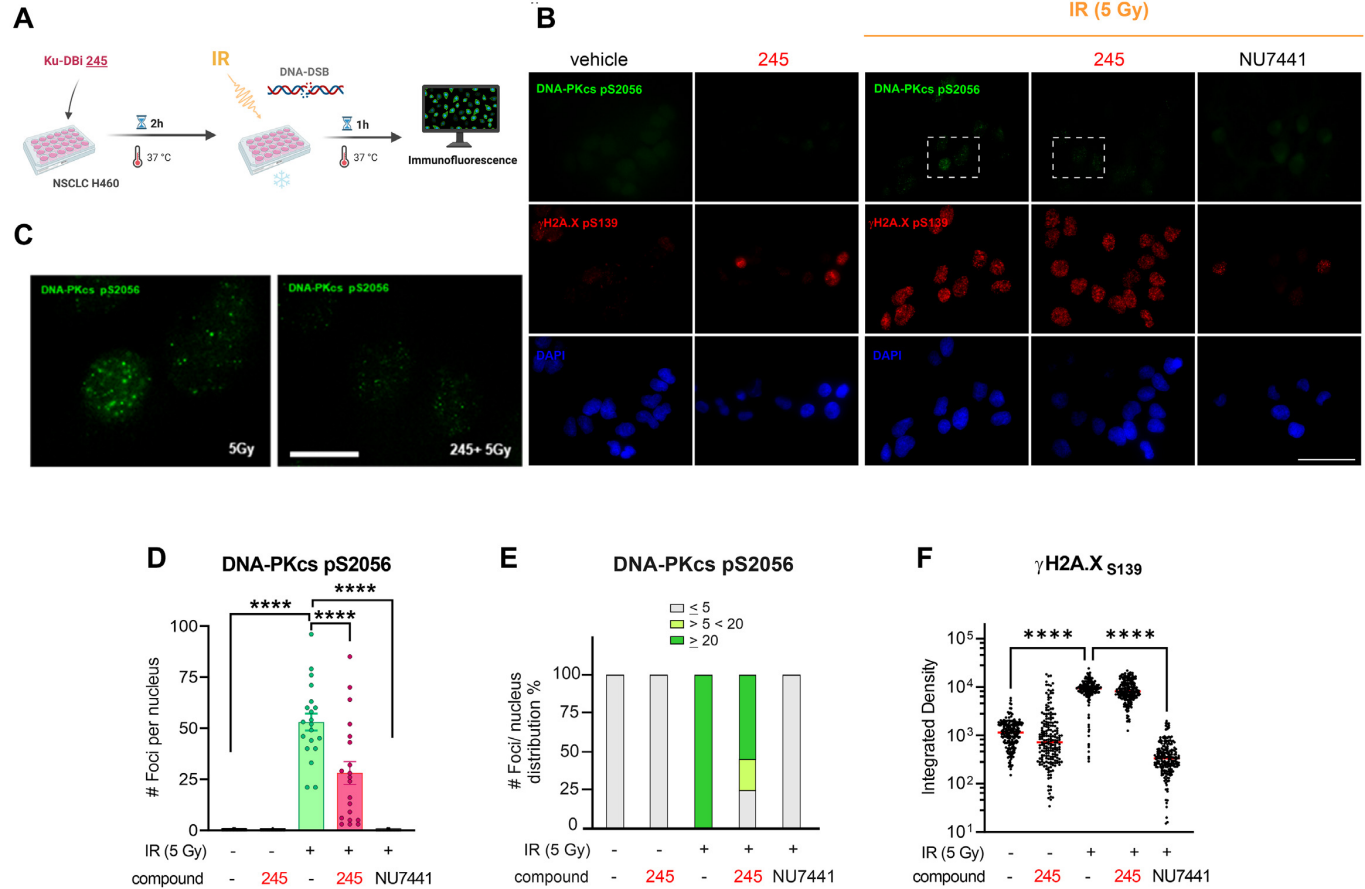
Impact of Ku-DBi's on the S2056 autophosphorylation in response to ionizing radiation. (**A**) Schematic representation of the treatment, created in *BioRender.com*. H460 cells were incubated for 2 h with Ku-DBi **245** followed by IR and fixed 1 h after treatments. (B) IF staining of DNA-PKcs p(S2056) (green) and γ-H2AX (pS139) (red) following treatment. Sb: 10 μm. (**C**) 60× images magnification from 5 Gy (IR) and Ku-DBi **245**+ IR conditions (insets in panel **B**), Sb: 2.5 μm. (**D**) Quantification of integrated fluorescence intensity of γ-H2AX foci. (E) Quantification of DNA-PKcs foci. (**F**) Frequency of distribution of DNA-PKcs foci per nucleus. Data are presented as the mean and SEM of triplicate determinations. ***P <0.0001 as calculated by one-way ANOVA with Tukey's multiple comparisons test.

**Figure 5.**
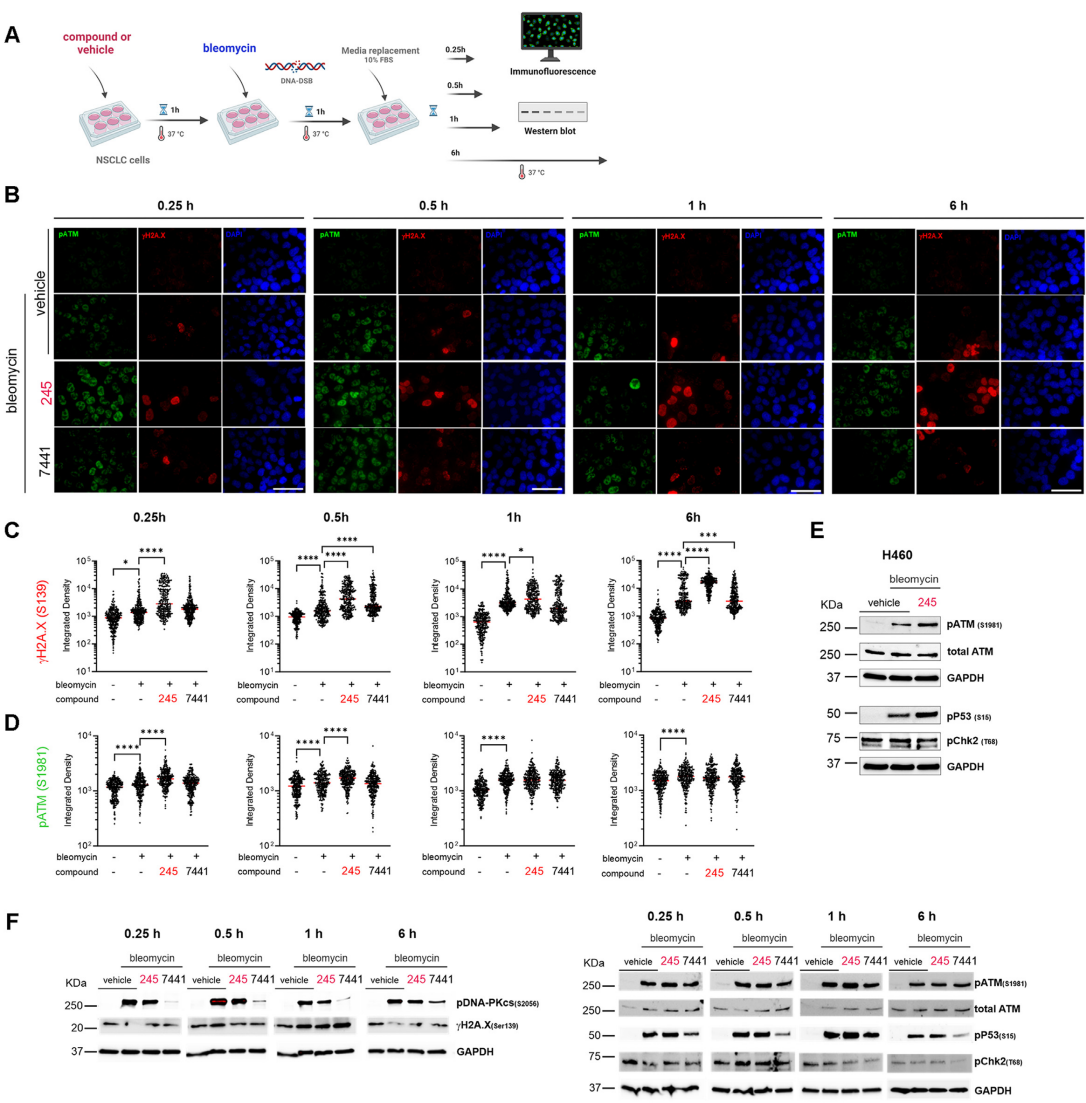
Impact of novel Ku–DNA binding inhibitors on the ATM DDR pathway in NSCLC cells. (**A**) Schematic representation of treatments used in this assay. NSCLC cells were pre-incubated with 20 μM Ku-DBi **245** and 1 h 10 μM bleomycin 10 μM **NU-7441** or vehicle. Cells were fixed for IF detection 0.25, 0.5, 1 or 6h after treatments were ended, and proteins were extracted for Western blot immunodetection. (**B**) IF staining of ATM p(S1981) (green) and γ-H2AX (pS139) (red) following treatments. Sb: 10 μm. (**C**) Quantification of integrated fluorescence intensity of γH2AX foci and (**D**) ATM (pS1981). Data are presented as the mean and SEM of triplicate determinations. (**E**) H460 cells were pre-treated with 20 μM Ku-DBi **245** or vehicle. DNA damage was induced by bleomycin treatment and cells were processed for immunodetection at 0.5h after treatment was ended. (**F**) Western blot analysis from A549 cells extracts. ****P <0.0001, *P <0.05 as calculated by one-way ANOVA with multiple comparisons test.

In addition, immunofluorescence staining revealed that irradiated cells showed a DNA-PKcs foci punctate pattern that was different from the observed homogeneously distribution of fluorescence intensity in the DNA-PKcs foci formed after 1 h bleomycin treatment (Figure 3A). We next quantified the number of DNA-PKcs foci contained in each cell nuclei. This analysis allowed us to calculate that 50% of the analyzed cells that were exposed to 5 Gy IR showed >20 foci per nucleus, compared to 85% of cells that were exposed to higher doses of IR (10 Gy), which contained >20 foci per nucleus (Figure 3F and G). Having established the conditions and parameters for combination treatments we next determined the effect of our Ku-DBi on the cellular response to IR. Based on our previously reported data on potency, specificity, kinase activity inhibition and solubility, in addition with our results shown above we chose Ku-DBi **245** as a representative compound for further experiments. H460 cells were pre-treated for 2 h with 20 μM Ku-DBi **245** and irradiated with 5 Gy (Figure 4). Cells were allowed to recover for 1 h at 37°C before fixation for IF detection of phospho-DNA-PKcs (S2056) and γ-H2AX (S139) (Figure 4A). A reduction of DNA-PKcs (S2056) was observed when irradiated H460 cells were pre-incubated with Ku-DBi **245** (Figure 4B, C, S1). However, quantification of DNA-PKcs foci (Figure 4D), and the analysis of frequency of distribution of DNA-PKcs foci per nucleus (Figure 4E) showed that Ku-DBi **245** pre-treatment followed by IR induced a significant reduction in the number of DNA-PKcs foci per nucleus, along with a reduction close to 50% of the number of cells that showed more than 20 foci per nucleus, consistent with western blot analysis (Figure S1). Although a modest reduction in the fluorescence intensity of γ-H2AX foci in Ku-DBi **245** pre-treated cells was observed, this difference is not significant (Figure 4F). This result is different from the observations with bleomycin (Figure 2C) where a decrease in γ-H2Ax was observed immediately after bleomycin treatment. The difference could be the result of either the different treatment modalities or the 1 h post DNA-damage incubation time. A time-course assay was employed to determine the temporal effect of DDR signaling and the impact of Ku inhibition. The experiment depicted in Figure 5A involved pre-treating H460 cells with vehicle of 245 for 1 h followed by the addition of bleomycin for 1 h. Following the 2 h total incubation time, drug containing media was removed, cells were washed, and fresh media added. Cells were then processed for IF from at 15, 30 and 60 min and 6 h of recovery time. The IF data and quantification presented in Figure 5B and C demonstrate that an increase in γ-H2AX is observed in as a function of time after bleomycin/245 combination treatment. This data is consistent with the IR data in Figure 4 and suggests that the decrease in γ-H2AX observed upon immediate processing of cells ‘during’ with no recover period is reversible with increasing incubation time after treatment.

### Ku–DNA binding inhibition impacts the ATM DDR signaling pathway in NSCLC cells

Having established that blocking the Ku–DNA interaction impacts the cellular response to DNA-DSB inducing agents via a reduction in DNA-PKcs autophosphorylation with differential effect on γ-H2AX levels, we sought to further probe impacts on the DDR. Previous evidence has shown that DNA-PK also plays a role in the larger DDR, phosphorylating ATM and a large number of other downstream targets to promote efficient and accurate DNA repair, or becoming phosphorylated by ATM at T2609 in response to DNA damage (11); (32). In addition, ATM has been described to possess autophosphorylation activity at S1981, which can activate the kinase by promoting dissociation of the inactive dimer to active monomers in response to DNA damage (13). This model is somewhat confounded by demonstration of redox regulation of ATM where activation involved intermolecular disulfide driven dimer activation (33). The potential for this crosstalk between DNA-PKcs and ATM described in the literature led us to interrogate how the DNA-PKcs abrogation via the inhibition of Ku–DNA binding impacts ATM phosphorylation and the ATM-dependent signaling pathways as a function of Ku-DBi treatment followed by the induction of DNA-DSB. Increase in ATM autophosphorylation at S1981 was observed in response to bleomycin at all time points post treatment. Phosphorylation increased at 15 and 30 min post DNA damage treatment, suggesting a time-dependent activation under Ku–DNA binding inhibition followed by DNA-DSB induction conditions (Figure 5D). These results were confirmed by analysis of integrated fluorescence intensity of pATM (S1981) foci, demonstrating the significant increase in the pATM levels for the combined treatment of Ku-DBi+ bleomycin compared to the bleomycin-only control. We observed that ATM activation was sustained from 0.25 h until at least up to 1 h after bleomycin was removed. On the other hand, a longer sustained increase for γ-H2AX was observed at least up to 6 h after the 1 h bleomycin treatment ended (Figure 5C). These results were confirmed in Western blot analysis of protein extracts from cells treated which showed increased levels of ATM autophosphorylation at S1981 in response to bleomycin. This was further increased in cells treated with the combination (Ku-DBi + bleomycin) (Figure 5E). In addition, we did not detect noticeable changes in the levels of DNA-PKcs phosphorylation at T2609 when cells were treated with Ku-DBi and DSB-inducing agent compared to the controls, which suggests this increase in the ATM phosphorylation events would not affect the DNA-PKcs phosphorylation mediated by ATM autophosphorylation (data not shown). Considering the observation of S1981 autophosphorylation and its role in ATM activation, we examined the downstream effectors p53 (pS15) and Chk2 (pT68). The phosphorylation of p53 was increased by bleomycin treatment and further increased by **245** combination treatment (Figure 5E). Treatment with **245** alone had no effect on pATM, p53 or pChk2 (data not shown). Interestingly p-Chk2 was easily detected in vehicle treated cells and was largely unaffected by bleomycin, **245** or the combination (Figure 5E).

Consistent with these observations, western blot results from A549 cell extracts confirmed the reduction of DNA-PKcs phosphorylation levels at S2056 under combined treatment conditions (Figure 5F). Impact on γ-H2AX were more variable over time as was ATM autophosphorylation. A modest increase in the combination was observed at 30 min which was further enhanced at 60 min. A similar temporal regulation of p-p53 was observed with maximal increase in the combination observed at 60 min recovery time post treatment. Phosphorylation of Chk2 was again largely unaffected by bleomycin, **245** or the combination treatment at all time points analyzed.

### Inhibition of Ku-DNA binding impacts the cellular viability in NSCLC cell lines harboring ATM and p53 alterations

**Figure 6.**
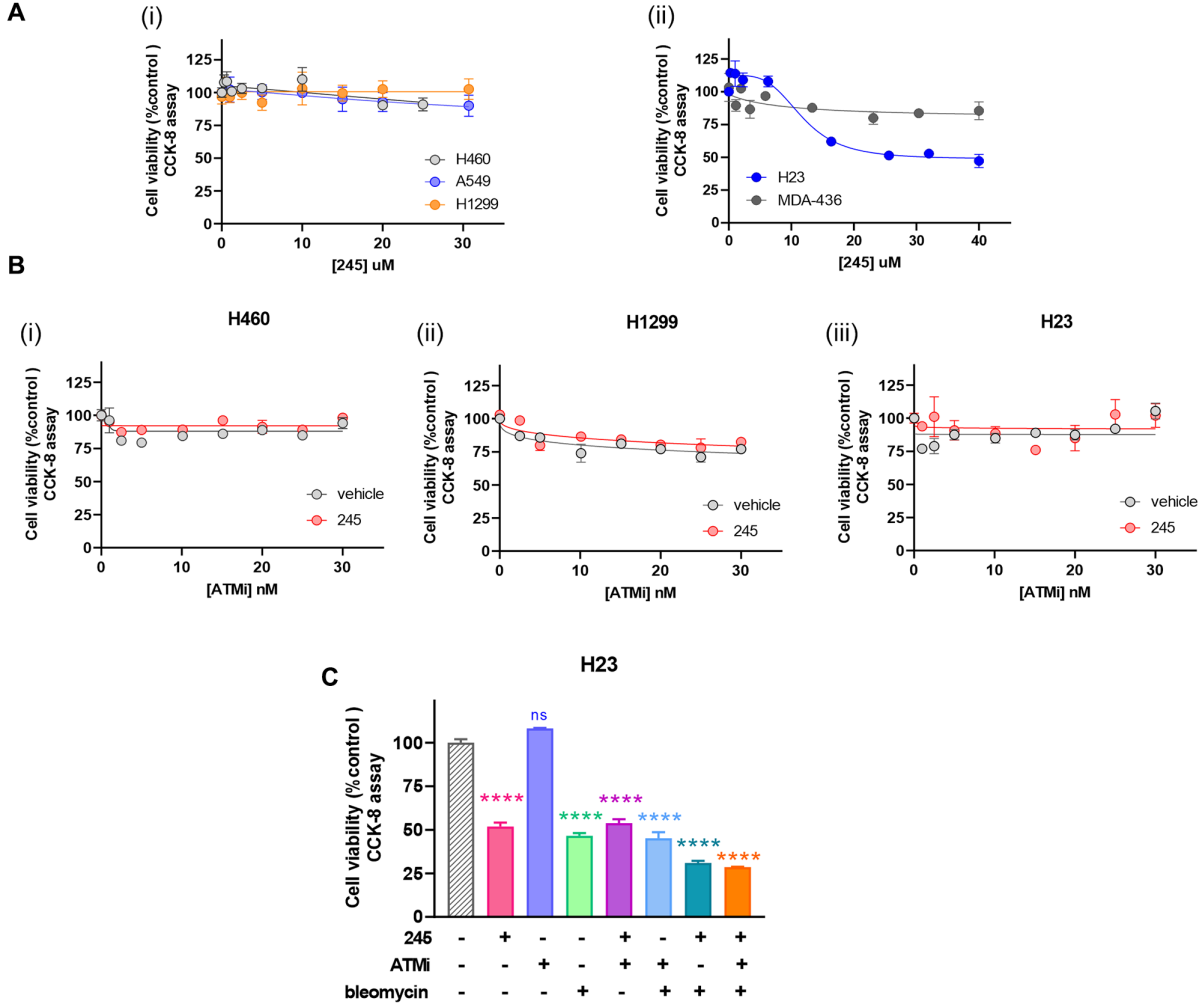
Ku-DBi's differentially impact on NSCLC cell lines carrying different ATM, p53 and BRCA1 genetic status. (**A**) Effect of Ku-DBi **245** as a single agent in cells carrying mutations for ATM, p53 and BRCA1. H460 (ATM wt, p53 wt), A549 (ATM wt, p53 wt) and H1299 (ATM wt, p53 deficient) (i), and H23 (ATM deficient, p53 mutated) or BRCA1 mutation MDA-436 (ATM wt, p53 mutated) (ii) were treated with increasing concentrations Ku-DBi **245** for 72 h and cellular viability was determined by CCK-8 assay. Data are presented as the mean and SEM of triplicate determinations. (**B**) H460, H1299 and H23 cells were treated with 20 μM Ku-DBi or vehicle and increasing concentrations of the **AZD0156** ATM inhibitor. Cells were incubated for 72 h and cellular viability was determined by CCK-8 assay. Data are presented as the mean and SEM of triplicate determinations. (**C**) H23 cells were pre-incubated with 20 μM Ku-DBi, 30 nM **AZD0156**, 1 μM bleomycin or vehicle or combinations. Cells were incubated for 72 h after which cell viability was determined by CCK-8 assay. Data are presented as the mean and SEM of triplicate determinations. ****P < 0.0001 as calculated by one-way ANOVA with Tukey's multiple comparisons test.

In light of the observation that inhibition of Ku–DNA binding leads to differential modulation of DDR signaling via ATM activation driven by autophosphorylation events in conjunction with p53 phosphorylation, we assessed how Ku-DBi's impact cells carrying mutations for ATM, p53 and BRCA1 (Figure 6). We selected four NSCLC lines, NCI H460 a large cell carcinoma and A549 adenocarcinoma which are both wildtype for ATM and p53, and H23 adenocarcinoma that is ATM-deficient and possess the M246I p53 mutation which results in low p53 protein expression levels and loss of function (34,35). We also included the H1299 NSCLC cell line that is ATM wild type and p53 null and the MDA-MB-436 breast cancer cell line which is ATM wild type, BRCA1 deficient and harbors a p53 frameshift mutation that results in low protein expression (36). Cells were plated and treated with increasing concentrations of Ku-DBi **245** for 72 h after which cell viability was determined and the results showed that Ku-DBi's single-agent activity is only observed in the ATM deficient H23 cell line (Figure 6A). That the MDA-MB-436 are relative insensitive to Ku-DBi's suggests that the reduction in p53 is not impacting sensitivity and it is the loss of ATM. The insensitivity of the H1299 which are p53 null also support this conclusion. Considering the sensitivity of ATM null cells, we sought to determine if pharmacologic inhibition of ATM would sensitize cells to Ku-DBi treatment. The data demonstrate that in H460 and H1299 cells, Ku inhibition does not impact the ATM inhibitor sensitivity as the cells remain unaffected (Figure 6B, panel i and ii) and no impact was observed in the H23 ATM deficient cell line as was expected (Figure 6B, panel iii). We then assessed whether it is possible to sensitize ATM null cells to DSB-inducing agents. We examined the effect of the combined treatment of Ku-DBi and bleomycin on the cellular viability in the NSCLC H23 ATM-deficient cell line (Figure 6C). NSCLC H23 cells were pre-incubated for 24 h with 20 μM Ku-DBi, or vehicle, and then treated with 1 μM bleomycin. Cells were incubated for 72 h in total after which cellular viability was determined. The results demonstrated Ku–DNA binding inhibition induced by Ku-DBi increases the sensitivity of the H23 ATM-deficient cell line to bleomycin. These data are indicative of at least an additive interaction of bleomycin and Ku-DBi **245** and the effect of **245** in greater in the ATM deficient cells (Figure 6C) compared to H460 ATM wildtype (Figure 1E). These results are consistent with previous literature where a connection between the p53 activation and cellular death is associated with the activation of programmed cell death pathways (37) and also consistent with the DNA-PK inhibitors increased potency in ATM deficient cell lines (24).

## DISCUSSION

The DNA-PKcs/Ku interaction is involved in two aspects of DNA metabolism that are critical in cancer development and the response to cancer therapy: NHEJ-catalyzed repair of DNA DSB (9) and the larger DDR, both of which are essential to ensure genome stability (4). Despite advances in understanding the molecular mechanisms of DNA-PK activation, current DNA-PK inhibitors have been mainly developed to target the catalytic subunit of DNA-PK, DNA-PKcs. In our previous work, we interrogated a novel strategy to specifically block the Ku–DNA interaction towards the development of a new class of DNA-PK inhibitors (Ku-DBi's) (25). In the present study, we assessed the cellular effects of these Ku-DBi's and their impact as single agents or in combination with DNA-DSB inducing agents on the DDR pathways. Despite the high protein binding and limited cellular uptake of these current inhibitors, we have demonstrated an alteration on the DDR in response to the chemical blockade of the Ku–DNA interaction. We observed abrogation of DNA-PKcs autophosphorylation events at S2056 but no effect on T2609 phosphorylation in response to DSB-inducing agents. These data are consistent with previous reports demonstrating that T2609 is phosphorylated by ATM (38).

In addition, Ku-DBi-bleomycin treatment resulted in a decrease in γ-H2AX levels, at early time points after treatment compared to bleomycin treatment alone. This suggests an inhibition of DDR; however, the analysis of later times in both H460 and A549 NSCLC cells revealed an increase in γ-H2AX levels in Ku-DBi treated cells compared to controls. These data suggest that Ku-DBi's temporally impact DDR activation and H2AX phosphorylation is likely being catalyzed by DNA-PK and ATM differentially throughout the time course of this study. Interestingly, our observations from the time-dependent ATM phosphorylation assessments under Ku–DNA binding inhibition conditions, showed an increase in the ATM autophosphorylation events at S1981 which correlates with γ-H2AX levels. It is therefore likely that H2AX could be phosphorylated mainly from ATM at later time point after break induction in the presence of DNA-PK inhibition, which is also consistent with literature (39). Moreover, we observed a time-dependent increase in the ATM phosphorylation levels in NSCLC cells treated with Ku-DBi's, consistent with the ATM hyperactivation described in the absence of DNA-PK kinase activity in human cells (32). These data suggest that abrogation of DNA-PKcs autophosphorylation at S2056 induced by Ku-DBi's could be exerting an effect on the activation of ATM via autophosphorylation establishing an inverse relationship between DNA-PKcs and ATM. This differential activity could then contribute to the observed increase in p53 phosphorylation and induction of reduction of cellular viability leading to the increased sensitivity to DSB in Ku-DBi treated cancer cells.

Moreover, we demonstrated that Ku chemical inhibition differently impacts cells with distinct genetic backgrounds carrying ATM and/or p53 alterations. Only modest anticancer activity is observed in ATM and p53 wt NSCLC cells while ATM loss in a p53 mutant background showed the greater effect of Ku-DBi's. Interestingly, p53 loss results in no effect of Ku-DBi's as evidenced by the H1299 NSCLC cell line. Together, these results support the use of Ku-DBi to sensitize ATM-deficient cells that carry p53 mutations. In this context, it becomes tempting to extend our studies on the utility of Ku–DNA binding inhibitors in other genetic backgrounds beyond ATM and p53.

Considering the DNA-PK-independent role of Ku in DNA metabolism, it is likely that other genetic alterations in the DDR could impact sensitivity to Ku-DBi's. Ku–DNA binding inhibition could play an effective role in DNA-PK independent-Ku mechanisms, like telomere metabolism and maintenance where Ku has been demonstrated to support telomerase catalyzed telomere length maintenance (40,41). One could then envision less Ku-DBi sensitivity in cells that maintain telomeres via the alternative lengthening of telomeres (ALT) pathway compared to telomerase positive cancers (42,43).

With catalytic active site DNA-PK inhibitors making their way into clinical trials, the potential for drug resistance remains a concern as has been demonstrated with many other kinase targets (44). One mechanism of kinase inhibitor resistance is an active site mutation that abrogates inhibitor binding (45,46). The utility of Ku-DBi's is a potential strategy to overcome the resistance phenomenon to DNA-PK inhibitors where continued pathway inactivation would provide clinical benefit. One could even envision a combination approach where catalytic DNA-PK inhibitors and Ku-DBi's would be individually employed in alternating cycles of treatment to maintain pathway inactivation without continued chronic dosing of one individual agent. Importantly, the data presented in this work supports the application of chemical Ku–DNA binding inhibition to abrogate DNA-PKcs autophosphorylation, impact DSB repair, modulate DDR signaling, and ultimately impacting cancer cell death, and thus is a promising approach as part of an anticancer therapeutic strategy in combination with DNA DSB-inducing agents.

## Supplementary Material

zcad003_Supplemental_Files
