# Supplementary material for: Ku–DNA binding inhibitors modulate the DNA damage response in response to DNA double-strand breaks
Source: NAR Cancer. 2023 Feb 6;5(1):zcad003. doi: 10.1093/narcan/zcad003 (PMC9900423; doi:10.1093/narcan/zcad003)

SUPPLEMENTARY INFORMATION

Ku-DNA binding inhibitors modulate the DNA damage response in response to DNA double-strand breaks

Pamela L. Mendoza-Munoz[1], Navnath S. Gavande[2,3], Pamela S. VanderVere-Carozza[1], Katherine S. Pawelczak[4], Joseph R. Dynlacht[5], Joy E. Garrett[5] and John J. Turchi[1,4,6,*]

[1] Department of Medicine, Indiana University School of Medicine, Indianapolis, IN, 46202, USA.
[2] Department of Pharmaceutical Sciences, Wayne State University College of Pharmacy and Health Sciences, Detroit, MI, 48201-2417, USA.
[3] Molecular Therapeutics Program, Barbara Ann Karmanos Cancer Institute, Wayne State University, Detroit, MI, 48201-2417, USA.
[4] NERx Biosciences. Indianapolis, IN, 46202, USA.
[5] Department of Radiation Oncology, Indiana University School of Medicine, Indianapolis, IN, 46202, USA.
[6] Department of Biochemistry and Molecular Biology, Indiana University, School of Medicine, Indianapolis, IN, 46202, USA.

* To whom correspondence should be addressed. Tel: +1 317 278 1996; Fax: +1 317 274 0396; Email: jturchi@iu.edu.

Table S1. Ku- DNA binding inhibition does not affect the short-term cellular viability. Logistic growth. YM, maximum population (Confluence %); Y0, starting population (Confluence %); k, rate constant (day^{-1}); Xint, X coordinate of the first inflection point.

$$Y=YM*Y0/((YM-Y0)*exp(-k*x) +Y0)$$

Compound	YM	Y0	k	Xint
**1%DMSO**	89.6	11.7	0.86	1.2
**NG-245**	78.7	12.2	0.81	1.2
**NG-149**	84.4	13.4	0.78	1.3
**NG-322**	80.2	13.2	0.82	1.3

Movie S1. 7 days Incucyte live cell imaging recording for NSCLC H460 cells treated with vehicle.

Movie S2. 7 days Incucyte live cell imaging recording for NSCLC H460 cells treated with 20 μM Ku-DBi 245.

Movie S3. 7 days Incucyte live cell imaging recording for NSCLC H460 cells treated with 20 μM Ku-DBi 245.

Movie S4. 7 days Incucyte live cell imaging recording for NSCLC H460 cells treated with 1 μM bleomycin.

Movie S5. 7 days Incucyte live cell imaging recording for NSCLC H460 cells treated with 20 μM Ku-DBi 245 and 1 μM bleomycin combination.

Figure S1. Impact of Ku-DBis on the S2056 autophosphorylation in response to ionizing radiation. H460 cells were incubated for 2h with 20 μM Ku-DBi 245, 10 μM NU-7441 or vehicle followed by IR and fixed 1h after treatments. Western blot analysis from H460 cells extracts.

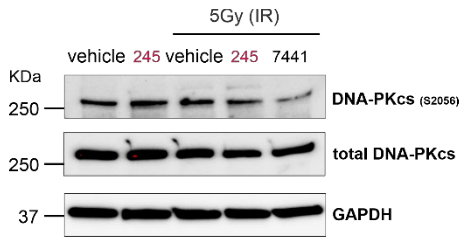

Supplement: zcad003_Supplemental_Files [file zcad003_supplemental_files.zip › 3. Supplemental data.pdf]
